# Non-Destructive Spatial Mapping of Glycosaminoglycan Loss in Native and Degraded Articular Cartilage Using Confocal Raman Microspectroscopy

**DOI:** 10.3389/fbioe.2021.744197

**Published:** 2021-10-28

**Authors:** Tianyu Gao, Alexander J. Boys, Crystal Zhao, Kiara Chan, Lara A. Estroff, Lawrence J. Bonassar

**Affiliations:** ^1^ Department of Materials Science and Engineering, Cornell University, Ithaca, NY, United States; ^2^ Sibley School of Mechanical and Aerospace Engineering, Cornell University, Ithaca, NY, United States; ^3^ Kavli Institute at Cornell for Nanoscale Science, Ithaca, NY, United States; ^4^ Meinig School of Biomedical Engineering, Cornell University, Ithaca, NY, United States

**Keywords:** confocal Raman microspectroscopy, articular cartilage, osteoarthritis, glycosaminoglycans, cartilage degradation model

## Abstract

Articular cartilage is a collagen-rich tissue that provides a smooth, lubricated surface for joints and is also responsible for load bearing during movements. The major components of cartilage are water, collagen, and proteoglycans. Osteoarthritis is a degenerative disease of articular cartilage, in which an early-stage indicator is the loss of proteoglycans from the collagen matrix. In this study, confocal Raman microspectroscopy was applied to study the degradation of articular cartilage, specifically focused on spatially mapping the loss of glycosaminoglycans (GAGs). Trypsin digestion was used as a model for cartilage degradation. Two different scanning geometries for confocal Raman mapping, cross-sectional and depth scans, were applied. The chondroitin sulfate coefficient maps derived from Raman spectra provide spatial distributions similar to histological staining for glycosaminoglycans. The depth scans, during which subsurface data were collected without sectioning the samples, can also generate spectra and GAG distributions consistent with Raman scans of the surface-to-bone cross sections. In native tissue, both scanning geometries demonstrated higher GAG content at the deeper zone beneath the articular surface and negligible GAG content after trypsin degradation. On partially digested samples, both scanning geometries detected an ∼100 μm layer of GAG depletion. Overall, this research provides a technique with high spatial resolution (25 μm pixel size) to measure cartilage degradation without tissue sections using confocal Raman microspectroscopy, laying a foundation for potential *in vivo* measurements and osteoarthritis diagnosis.

## 1 Introduction

Osteoarthritis (OA) is a degenerative disease that mainly affects articular cartilage and related joint tissues. Articular cartilage is the tissue at the end of long bones that provides a smooth surface and lubricated joint motion, as well as a mechanically robust structure for load bearing. Structurally, articular cartilage is composed of three layers: a thin surface layer that contains a dense collagen fiber network that is oriented parallel to the articular surface and with low proteoglycan content; a middle zone that has relatively disorganized collagen fibers and a higher proteoglycan content; and, adjacent to the bone, a deep zone that contains collagen fibers oriented perpendicular to the bone surface and has the highest proteoglycan content (Muir et al., 1970). During the progression of OA, the major organic components of cartilage extracellular matrix (ECM), the collagen network and proteoglycans, are gradually degraded by enzymes released during the inflammatory response (Mort and Billington, 2001; Martel-Pelletier et al., 2016). This degradation causes roughening of the articular surface, leading to mechanically induced ECM degradation and death of chondrocytes (cells that renew and maintain the ECM). Over time, the cartilage becomes severely eroded, causing thickening of subchondral bone and eventually direct bone-on-bone contact within the joint space. Clinically, OA is diagnosed by radiographic methods, such as Kellgren-Lawrence scores (Kellgren and Lawrence, 1957), in which joint space narrowing is considered the main diagnostic indicator. Notably, joint-space narrowing is indicative of advanced disease. As such, X-ray techniques cannot detect OA at its early stages. In early stage OA, the cartilage structure remains intact while chemical degradation of ECM components is happening close to the articular surface. The major hallmark for early stage OA is the depletion of subsurface glycosaminoglycans (GAGs), such as aggrecan (Lark et al., 1995). This work addresses the need for an analytical technique able to detect these chemical changes in cartilage composition that are indicative of early stage OA. Specifically, we use Raman microspectroscopy to spatially map the removal of proteoglycans from bovine cartilage.

Experimental studies of early-stage cartilage disease typically utilize histological stains to demonstrate cartilage quality (Pritzker et al., 2006). For instance, the scoring system presented by the Osteoarthritis Research Society International (OARSI) is fully developed based on compositional and structural information in histology sections, including proteoglycan reduction and surface discontinuity. Typical histology stains such as Safranin-O and Alcian Blue (for proteoglycans) or Picrosirius Red (for collagen) are utilized to demonstrate the distribution of the main biochemical components. These techniques are effective for qualitative assessment of the distribution of matrix components but are inherently not quantitative. Fourier transform infrared (FTIR) microspectroscopy has also been applied for more quantitative analysis of degraded cartilage. Concentrations of biomolecules can be measured quantitatively using FTIR, based on the absorption spectra of the tissue (Camacho et al., 2001; Rieppo et al., 2010; Khanarian et al., 2014). However, both histology and FTIR microspectroscopy require tissue removal and subsequent processing including fixation, dehydration, sectioning, and mounting. As such, their utility for clinical diagnosis and disease monitoring is limited. Magnetic resonance imaging (MRI) is used clinically for assessing cartilage quality (Alhadlaq et al., 2004; Goodwin et al., 2004; Potter et al., 2009; Bron et al., 2013). Although MRI collects information about thickness, surface characterization, and biochemical components, it is limited in spatial resolution (∼100 μm), which limits its ability to track the zonal compositional changes in the early stages of OA that are on the length scale of 150 µm (Glover and Mansfield, 2002).

Raman microspectroscopy is capable of measuring structural information non-destructively, with an adequate resolution (<1 μm) to collect signals related to biochemical composition and structure (Bergholt et al., 2016b). This method is based on reflective vibrational spectroscopy and has been applied to several kinds of tissue such as ligaments, cartilage, osteochondral junctions, or bones, of which the major components are quite limited (water, collagen, proteoglycans, minerals) (Morris et al., 2002; Raghavan et al., 2010a; Raghavan et al., 2010b; Takahashi et al., 2014; Boys et al., 2019; Das Gupta et al., 2020). Unlike FTIR, the Raman signal of water does not have a strong overlap with ECM components, and as such, this technique can be applied to hydrated or even submerged tissue (Gamsjaeger et al., 2014; Irwin et al., 2021). Raman microspectroscopy has been applied to cartilage samples resulting in composition maps with a ∼0.3 μm spatial resolution. Chondrocytes are visible and the distribution of major components of cartilage (water, collagen, GAG, cytoplasm, DNA) can be detected (Bergholt et al., 2016b). The normalized values of each component in Raman maps are also quantitatively related to the absolute biochemical concentration from the articular surface to the deep zone of the cartilage (Albro et al., 2018). However, most studies using Raman microspectroscopy were performed on healthy cartilage samples. The ability of Raman microspectroscopy to measure biochemical distributions within degraded or damaged cartilage has received much less attention. In previous work, mapping of the depth-dependent composition of cartilage was accomplished by sectioning the tissue and scanning from the articular surface to the bone (Bergholt et al., 2016b; Albro et al., 2018). While this technique is quantitative, sectioning the tissue clinically from a patient for compositional analysis can cause secondary damage to the osteoarthritic joint.

The objective of this study is to examine the effectiveness of confocal Raman microspectroscopy for mapping the composition of degraded cartilage. To achieve this objective, two scanning geometries were applied: 1) scanning from the cut face (referred to as cross-section scan), which enables quantification throughout the tissue depth, but cannot be accomplished clinically; 2) scanning confocally at the articular surface (referred to as depth scan), which has a limited depth of penetration, but could be applied *in vivo*. To assess the ability of confocal Raman microspectroscopy to image GAG loss from the articular surface, we used an established model of trypsin-induced degradation (Bonassar et al., 1995; Griffin et al., 2014; DiDomenico et al., 2019). We assessed the extent to which GAG distribution maps achieved by this confocal Raman microspectroscopy technique are comparable to traditional methods such as histology. The outcomes of this study will provide new methods and data for the development of confocal Raman microspectroscopy as a technique for non-destructive, high-resolution cartilage compositional analysis.

## 2 Materials and Methods

### 2.1 Cartilage Sample Preparation and Trypsin Degradation

**FIGURE 1:**
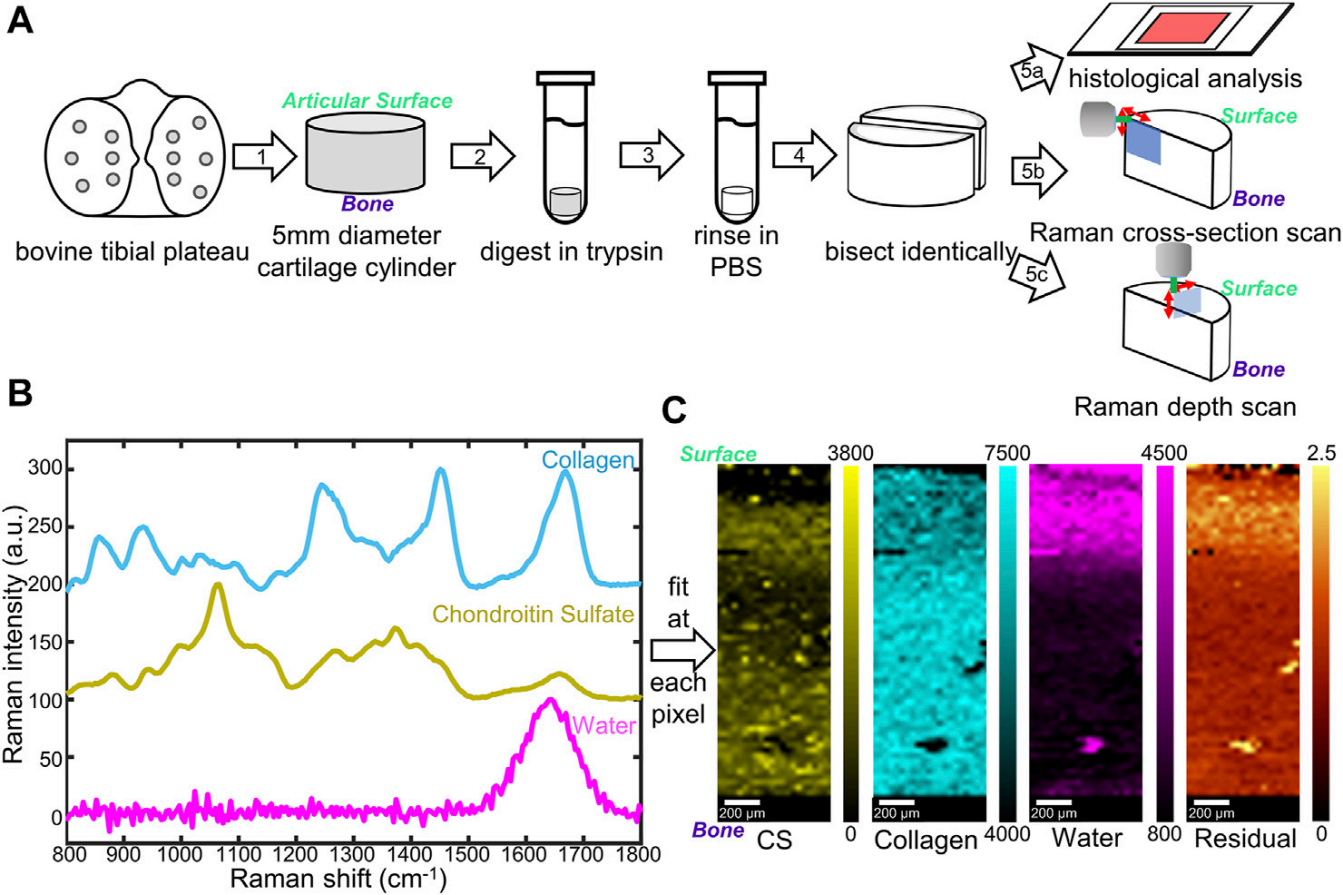
**(A)** Schematic diagram of sample preparation and characterization. Cylindrical cartilage samples (5 mm) were collected from a bovine tibial plateau (1), digested in trypsin (2), rinsed (3), and bisected (4) for histological stains (5a) as well as Raman microspectroscopy (5b, c). Two Raman scanning geometries, cross-section scan (5b) and depth scan (5c), have been applied. **(B)** Reference Raman spectra collected for individual components in cartilage: top (blue) lyophilized rat tail collagen, middle (yellow) shark cartilage chondroitin sulfate (CS) powder; bottom (magenta) deionized water. **(C)** Representative Raman coefficient maps, and residual generated by fitting the three reference spectra in **(B)** to spectra obtained from native cartilage samples.

Cartilage samples were harvested from neonatal bovine tibial plateaus (six animals) acquired from an abattoir (Gold Medal Packing, Rome, NY). Cylindrical cartilage samples were harvested by applying 5 mm biopsy punches to the articular surface of the contact zone between the tibial plateau and the femoral condyle to obtain full-thickness samples (Figure 1A, Step 1).

Cylindrical cartilage samples were randomly divided into three different groups: native group, fully digested group, and partially digested group. For the native group, samples were incubated in phosphate buffered saline (PBS, Invitrogen, Grand Island, NY) for 2 h. For the fully digested group, samples were incubated in a 0.25 wt% trypsin solution (lyophilized powder from bovine pancreas, ≥10,000 BAEE units/mg protein, Sigma-Aldrich, St. Louis, MO) for 2 h to completely remove proteoglycans (Bonassar et al., 1995). For the partially digested group, samples were incubated in a 0.000625 wt% trypsin solution for 0.5 h. All the samples were then rinsed in 10 mL PBS at 4°C for 1 h. Each sample was cut with a razor blade into two semi-cylindrical pieces, one was used for Raman analysis, the other was used for biochemical analysis.

### 2.2 Raman Data Collection

#### 2.2.1 Sample Preparation

All cartilage samples were anchored in 2 wt% low melting point agarose gel (gelling temperature 25 ± 5°C, Fisher Bioreagents) in Petri dishes (diameter 40 mm, height 12 mm) to prevent movement during confocal Raman mapping. The gel solution was preheated to 80°C in a water bath then cooled until 35°C before added to the Petri dishes. For tissue anchoring, a thin layer of the gel solution was added to each Petri dish at 35°C. Cartilage samples were then partially submerged in the solution, with the measuring surfaces exposed and facing upwards. The measuring surface of a sample is the articular-surface-to-bone cut face for the cross-section scan and is the articular surface for the depth scan. The dishes were cooled at 4°C until completely gelled. PBS was then added into the dishes to submerge the tissue samples.

#### 2.2.2 Raman Mapping

For scanning at the cross-section (*cross-section scan*), samples were mounted in the confocal Raman microscope to enable measurements along the articular surface-to-subchondral bone cut face of the sample (Figure 1A, Step 5b). The pixel size for cross-section scans was 30 × 30 μm.

For scanning at the articular surface (*depth scan*), samples were mounted in the confocal Raman microscope to enable measurements along the articular surface of the sample (Figure 1A, Step 5c). Depth-dependent information was collected by changing the working distance between the objective and the articular surface. The pixel size for depth scans was 25 × 25 μm.

All the confocal Raman spectra were collected with a WITec Alpha300R Confocal Raman microscope through a 40x dipping lens. A 532 nm green laser with a 62 mW power was used as the excitation source.

#### 2.2.3 Spectral Analysis

Using the WITec Project FIVE software (Version 5.2 PLUS, WITec, Ulm, Germany), spectra collected from cartilage samples were baseline subtracted via the Shape function of the WITec Project FIVE software (Shape size parameter: 400) and cropped to 800–1,800 cm^−1^ fingerprint area, then normalized to the maximum intensity of their –CH_2_ bending peaks (∼1,445 cm^−1^). The mineralized region close to the subchondral bone was confirmed by calculating the 906–986 cm^−1^ phosphate peak areas and masked out manually.

Three different constituents, collagen (Col), chondroitin sulfate (CS), and water, were chosen as standards for the analysis. Collagen from rat tail tendons was extracted, purified, and lyophilized as described previously (Iannucci et al., 2019), as the collagen standard sample. Chondroitin sulfate powder from shark cartilage (Sigma-Aldrich) was used as the CS standard. Deionized purified water was used as the water standard. For each reference, 12 Raman spectra were collected using a 532 nm laser at 62 mW through a 50× objective. Spectra were baselined as described above, cropped to 800–1,800 cm^−1^ fingerprint region, and normalized to their highest peaks (∼1,670 cm^−1^ for collagen, ∼1,060 cm^−1^ for CS, and ∼1,640 cm^−1^ for water). The average of the 12 spectra for each standard was considered as final reference spectra (Figure 1B).

The three reference spectra (Col, CS, and water) were used for non-negative fitting through the WITec True Component analysis. Briefly, the reference spectra were input through the Component Spectra Drop action, and fitting was performed using the linear combination based, Basis Analysis function. At each pixel, the spectrum collected from a sample was fit by linear combination of the three reference spectra. By amalgamating all the pixels, fitting coefficient maps, as well as residual images, were generated via WITec Project FIVE software.

### 2.3 Biochemical Analysis of Glycosaminoglycan Content

Dimethylmethylene Blue (DMMB) assay was applied to quantitatively measure the GAG content within the cartilage (Farndale et al., 1986). Cartilage samples (*n* = 8) were collected as described above. The first 1 mm to the articular surface and the last 1 mm to the bone were removed. Samples were randomly separated into two groups, each including four samples. One group was fully digested with trypsin, and the other group was treated with PBS (as described above). Both groups were then rinsed for 1 h, frozen overnight, and lyophilized for 48 h. The lyophilized samples were then subjected to biochemical analyses for GAGs using a method previously reported (Ballyns et al., 2008; DiDomenico et al., 2019). Briefly, samples were digested by 0.125 mg/mL papain (buffered aqueous suspension, Sigma-Aldrich) at 60°C for 16 h and mixed with 16 mg/L DMMB solution (pH = 1.5) in a well plate. The absorbance was measured at 525 nm using a plate reader (Biotek Synergy HT). GAG content of the sample was calibrated by comparing the absorbance of samples to the standard curve, which was determined by polynomial fitting of the absorbance data from GAG standards.

### 2.4 Histology

Semi-cylindrical cartilage samples were fixed in 10 v/v% formalin solution for 24 h, then in 70 v/v% ethanol for 24 h. Samples were embedded in paraffin and sectioned along the surface-to-bone cut face. Sections were dehydrated and deparaffinized using ethanol and xylene, then stained with Weigert’s hematoxylin for 10 min. For GAG visualization, the sections were counterstained with 0.001% fast green solution for 5 min and stained with 0.1% Safranin-O for 8 min. Slides were imaged with a Nikon Eclipse TE2000-S microscope (Nikon Instruments, Melville, NY) and a SPOT RT camera (Diagnostic Instruments, Steriling Heights, MI).

### 2.5 Statistical Analysis

To measure the statistical significance, quantitative data from the biochemical analysis were expressed as mean ± standard deviation. The pairwise comparisons of the biochemical data were analyzed *via* a one-way ANOVA test. Quantitative data from depth distribution measurements for native and digested cartilage were expressed as mean ± standard deviation. The pairwise comparisons were analyzed via mixed model analysis. Fitting coefficient data from cross-section scans and depth scans at different depths were analyzed through a mixed-model approach. Estimated values and confidence intervals were compared between different scanning geometries and between native and digested tissue. All analyses were performed using the IBM SPSS software platform.

## 3 Results

### 3.1 Raman Microspectroscopy of Native and Fully Digested Cartilage

To achieve a controlled amount of GAG degradation in cartilage samples, we used a trypsin digestion model to mimic the degradation in early stage OA (Figure 1A). Trypsin cleaves the core protein of aggrecan, the largest source of GAG in cartilage, but does not degrade the collagen helix (Bornstein et al., 1966; Anderson, 1969). In order to map the distribution of ECM components in trypsin-degraded bovine cartilage, confocal Raman mapping was applied along the surface-to-bone cut face of semi-cylindrical bovine cartilage samples. The main components (collagen, GAGs, and water) in all the samples, both native and fully digested, were mapped (700 µm by 2,200 µm) from the articular surface to the subchondral bone (Figure 1C).

**FIGURE 2:**
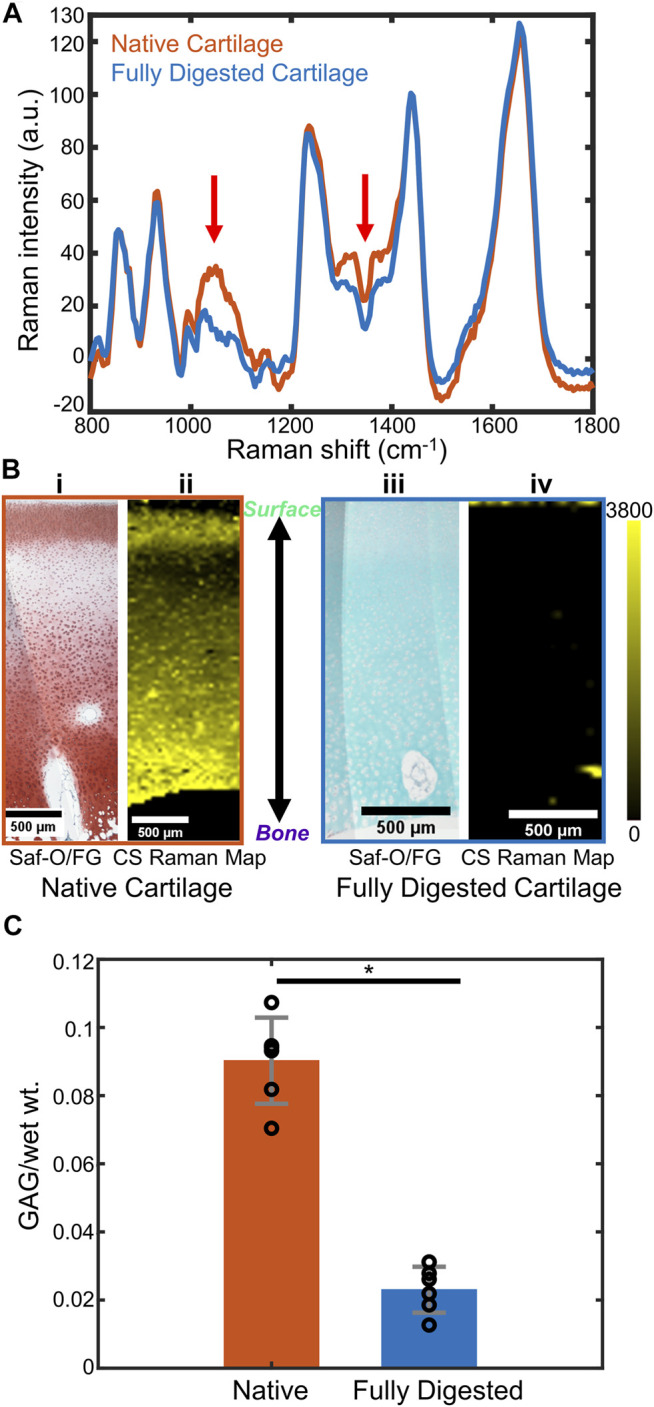
**(A)** Representative Raman spectra of native bovine cartilage and fully trypsin-digested bovine cartilage. (Spectra are obtained by averaging a 15-pixel by 15-pixel area at the center of the respective Raman images. Red arrows: GAG-related spectral regions). **(B) (i)** Safranin-O stained histology section of native cartilage, articular surface at the top of the image. **(ii)** CS coefficient map for the same region as **(i)**. **(iii)** Safranin-O stained histology section of fully trypsin-digested cartilage, articular surface at the top of the image. **(iv)** CS coefficient map for the same region as **(iii)**. **(C)** DMMB biochemical assay to determine the GAG content (GAG/wet wt.) of native cartilage and fully digested cartilage (*n* = 6, *: *p* < 0.01). Abbreviations: Saf-O, Safranin-O; FG, Fast Green; CS, chondroitin sulfate.

In a single spectrum taken from the middle zone of the native cartilage (Figure 2A), diagnostic peaks were observed in the 800–1,800 cm^−1^ fingerprint region, including ∼857 cm^−1^ proline peak, ∼935 cm^−1^ collagen triple helices, ∼1,060 cm^−1^ sulfate peak (from GAGs), ∼1,245 cm^−1^ amide III peak, ∼1,445 cm^−1^ -CH_2_ bending peak, and ∼1,668 cm^−1^ amide I peak (Pavlou et al., 2018). These peaks were also observed in the spectrum from fully digested cartilage, excluding the 1,000–1,200 and 1,300–1,450 cm^−1^ regions, which had a reduction of peak intensity compared to the spectrum of the native tissue. These regions were consistent with the main peaks of chondroitin sulfate (Figure 1B), indicating a reduction in the GAG content of the tissue.

The Raman maps were generated from the fitting coefficients for the reference spectra at each pixel and compared to Safranin-O stained histological sections. Safranin-O staining on native cartilage (Figure 2Bi) demonstrated a thin layer of cartilage with low GAG content at the articular surface and a relatively GAG-rich region in the deep zone. Similar distributions were observed in the Raman map of CS fitting coefficients (Figure 2Bii). For fully digested samples, however, the lack of pink-red Safranin-O staining throughout the section indicated the absence of proteoglycans in this sample (Figure 2Biii). The Raman map of the CS fitting coefficients showed a similar depletion of the GAG within the tissue (Figure 2Biv).

To quantitatively demonstrate the effect of the trypsin digestion on removal of GAGs from the cartilage samples, a DMMB biochemical analysis was performed on the bulk tissue (Figure 2C, *n* = 6 for each group). Based upon the DMMB assay, the fully digested cartilage demonstrated a ∼75% loss of total GAG components after trypsin digestion as compared to the native cartilage GAG content (*p* < 0.01).

### 3.2 Subsurface Confocal Raman Microspectroscopy of Native and Fully Digested Cartilage

**FIGURE 3:**
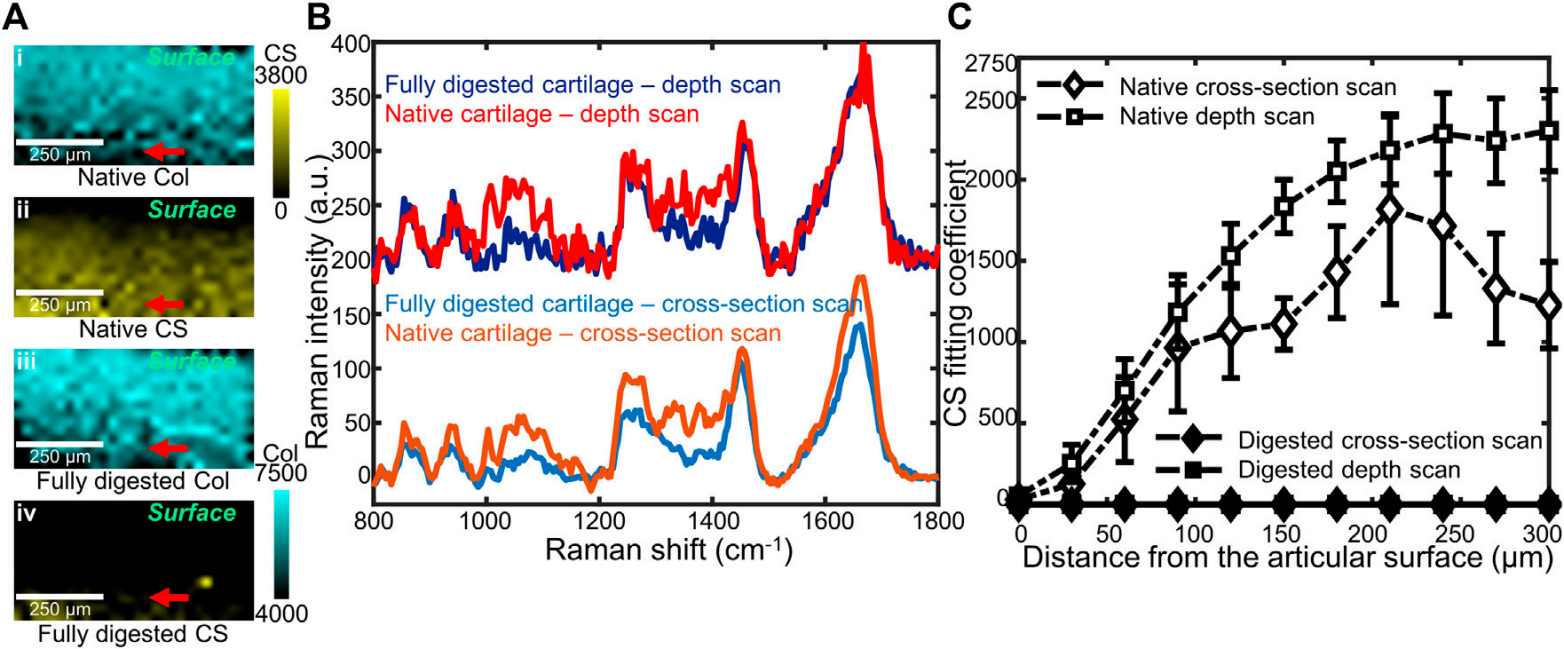
**(A)** Representative coefficient maps of CS and collagen (Col) obtained by confocal Raman depth scans on native (i,ii), and fully digested (iii,iv) articular cartilage. Red arrows: the 300 μm depth position at which spectra in **(B)** were obtained. **(B)** Representative Raman spectra of native cartilage and fully digested cartilage, all taken from 300 μm beneath the articular surface. Both cross-section scan and depth scan have been used, and a 200 a.u. offset was applied to each depth scan spectrum for clarity. **(C)** CS coefficient plotted as a function of distance from the articular surface, up to 300 µm beneath the surface. Data from two cross-section scans (native and digested) as well as two depth scans (native and digested) are plotted (*n* = 4).

Confocal Raman mapping was applied to native and fully digested tissue samples under the depth scan method (Figure 3A), in which depth-dependent information was collected confocally by moving the objective towards the articular surface and focusing into the tissue. For native tissue, the Raman map indicated a thin layer without CS at the articular surface of cartilage and a higher CS content at the deeper region. The Raman map of fully digested cartilage, however, demonstrated negligible CS content throughout the tissue. The collagen, on the other hand, is shown to be evenly distributed across the imaged area for both native and fully digested cartilage samples. Linear depth scans were performed starting from the articular surface and moving deeper into the tissue in 30 μm increments down to 300 μm. These scans were compared to those obtained by scanning at the cut face of cartilage from the surface to the deeper zone. Both scanning geometries were applied to native and fully digested tissue. Representative spectra at 300 μm below the articular surface collected via both geometries (Figure 3B) demonstrate similar spectra for native cartilage samples, showing that confocal spectra collection is possible within 300 μm of the surface. In fully digested cartilage samples, the spectra showed weaker signals in 1,000–1,200 and 1,300–1,450 cm^−1^ GAG regions and high similarity between the two different scanning geometries.

When the CS fitting coefficients are plotted as a function of distance from the articular surface (Figure 3C), in native tissue, a depth-dependent increase was seen when scanning below the surface (*p* < 0.01). Similar trends in GAG content of native tissue were observed by both scanning geometries: low at the articular surface and high at deeper regions (*p* = 0.111). For fully digested tissue, both scanning geometries show negligible GAG concentrations throughout the 300 µm scanning region.

### 3.3 Confocal Raman Mapping of Partially Digested Cartilage

**FIGURE 4:**
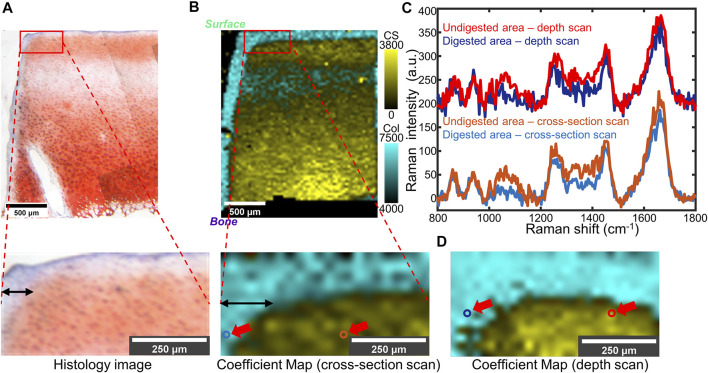
**(A)** Top: Light microscope image of partially trypsin-digested cartilage tissue stained by Safranin-O. Bottom: Higher-magnification of the area of interest (the red box). **(B)** Top: Overlaid coefficient map of CS and collagen took in the same region as **(A)**, taken by cross-section scan. Bottom: Higher magnification of the area of interest (the red box). **(C)** Point spectra collected from digested or undigested areas of the same sample. The collection points are shown as colored circles (indicated by red arrows) in (**B**, bottom) and **(D)**. A 200 a.u. offset was applied to each depth scan spectrum for clarity. **(D)** Overlaid coefficient map of the same sample as **(B)**, taken by depth scan. In **(B)** and **(D)**: Cyan: collagen; Yellow: CS.

To model early-stage OA in which GAG depletion occurs only near the articular surface, we used a much lower concentration of trypsin for a shorter digestion time. Confocal Raman mappings, both cross-section and depth scans, were applied to the partially digested bovine cartilage. Histological analysis with Safranin-O staining (Figure 4A) revealed a region of GAG depletion extending around 100 μm from the trypsin-exposed surfaces (Figure 4A, the top and left). The cross-section Raman scan showed a similar distribution (Figure 4B), with a ∼100 µm narrow band with high collagen coefficients but low CS coefficients observed at the trypsin-exposed surfaces. The CS coefficient map obtained from a depth scan recapitulated the distribution of collagen and GAGs observed in cross-section Raman maps and the histology image (Figure 4D). A curved interface between GAG-depleted and CS-rich regions was observed in all three analyses. The point spectra taken from the digested region and the undigested region demonstrate evident differences in the GAG-related regions (1,000–1,200 and 1,300–1,450 cm^−1^) (Figure 4C), which are consistent with the zonal differences observed in the histology images and the Raman maps.

## 4 Discussion

In this study, data collected from confocal Raman microspectroscopy were used to spatially map GAG loss in a trypsin digestion model of cartilage degradation. Cross-section Raman scans at the cut face of cartilage and confocal depth scans at the articular surface were applied to native and digested cartilage samples. The resulting maps successfully imaged the GAG distribution in partially degraded cartilage, a model for early stages of OA, with a pixel size of 25 μm.

Our research demonstrates that confocal Raman microspectroscopy has the potential to be utilized for compositional analysis of cartilage degradation. Although several analytical techniques have been utilized on tissues like cartilage which is made of only a few major components, they all have limitations. Traditional measurements for proteoglycan distribution in ECM are mostly performed via histological stains, such as Safranin-O or Alcian blue. However, fixation and sectioning of samples may lead to artifacts or inconsistencies between different sections. The staining outcome also varies among samples, making it unsuitable for quantitative analysis (Rosenberg, 1971; Király et al., 1996; Puchtler et al., 1988). Overall, despite being widely utilized in analyses of degraded cartilage, such methods reveal only qualitative distribution of each biochemical component within a sample. Spectroscopic techniques, like FTIR imaging, can yield more quantitative results (Rieppo et al., 2010). However, FTIR-based imaging techniques require specific sample preparation, including thin-sectioning, dehydration, optional embedding, and mounting on non-IR absorbing slides (Taylor and Donnelly, 2020). In contrast, Raman microspectroscopy can be utilized for quantitative analysis on thicker, unfixed, hydrated, or even submerged samples, because water does not have strong contributions to Raman signals, especially in the fingerprint region (Albro et al., 2018). Raman microspectroscopy has also been reported to be non-detrimental to cells (Gamsjaeger et al., 2014; Akiva et al., 2016; Bergholt et al., 2017), making it potentially available for analyzing tissue *in vivo* (Zeng et al., 2008; Duraipandian et al., 2014).

In this study, Raman spectra were collected via cross-section scans and depth scans. In cross-section scans, spectra were collected along the cut face of articular cartilage from the surface to the deep zone. Since the scans are at the cut face of the sample, cross-section scans can image the entire tissue from the articular surface to subchondral bone. For depth scans, however, spectra were collected confocally as optical slices of different depths by moving the objective and the focal plane. Spectral intensities decrease with depth into the tissue due to light absorption and scattering. However, these effects are similar across spectra, enabling quantitative comparisons by normalizing peaks of interest to reference peaks. Notably, these two scanning geometries yield similar results within the first 300 μm from the tissue surface (Figure 4). A previous study indicates that the superficial zone thickness is less than 250 μm for human, bovine, and canine cartilage (Panula et al., 1998; Quinn et al., 2013). Hence, obtaining reliable spectra from 300 μm beneath the articular surface enables detection of the GAG-rich region, where GAG depletion first occurs in early stage OA. Since the depth scan does not require cutting of the sample, it can be applied to intact tissue. In early stage OA, GAG depletion occurs at the surface layer of articular cartilage (Lark et al., 1995; Pritzker et al., 2006). Imaging from a 300 μm layer beneath the surface will be informative to assess early stage OA. Therefore, this technique has the potential to be applied under circumstances when tissue biopsies are saved or even through Raman-compatible arthroscopic probes (Bergholt et al., 2017).

In our study, both histology and biochemical analysis demonstrate a considerable GAG reduction in trypsin-digested tissue. Raman spectra and maps show similar results (Farndale et al., 1982; Chandrasekhar et al., 1987), with negligible coefficients of chondroitin sulfate detected, as indicated by near 0 values of CS fitting coefficients (Figures 2A,B). The results show that proteoglycan reduction can be measured by confocal Raman mapping. High-resolution Raman maps of GAG content recapitulate the distribution seen in histology. In native tissue, both geometries indicated low GAG concentration at the articular surface and an increase of GAG concentration at the deeper zone. In fully digested tissue both methods showed negligible GAG concentration throughout the samples. The distribution is consistent with previous work, in which the GAG distribution is determined by histology (Silverberg et al., 2014). The Raman coefficient maps, which showed a significant reduction in chondroitin sulfate content but no change in collagen content (Figures 3Ai,iii), are consistent with literature studies that show trypsin exposure removed proteoglycans but did not damage the collagen network (Bonassar et al., 1995; DiDomenico et al., 2019). Considering that the Raman spectral intensity of each component of ECM is assumed to be proportional to its concentration, this semi-quantitative method effectively compares the GAG distribution among samples with different levels of degradation (Figures 2Bii,iv; Figures 4B,D) (Albro et al., 2018).

The partially digested articular cartilage samples were used as a model for early-stage OA in which GAG depletion happens primarily near the surface but in the absence of surface fibrillation. Such degradation corresponds to OARSI grade 1 or 2 (Pritzker et al., 2006). In our study, Raman microspectroscopy detected GAG removal by trypsin degradation near the edge of samples in a thin band with a thickness around 100 μm. Notably, the resolution of Raman microscopy (25 μm pixel size) is better than other *in vivo* techniques like GAG-specific MRI. These Raman maps are also consistent with the Safranin-O staining of these tissues. Nonetheless, a slight difference in the thickness of the digestion edge was noticeable between Safranin-O staining (∼100 μm) and Raman maps (∼150 μm). This difference could be either due to the different sensitivity between Raman mapping and the Safranin-O dye or due to the deformation of histological samples under formalin fixation and sectioning.

While these results are promising, we still have some limitations for interpreting our data. For each sample, the time for spectral collection is long, typically for several hours. With the limitation of the confocal Raman microscope, spectra collected from regions more than 300 μm below the articular surface are noisy and unsuitable for reference fitting. A higher penetration depth might be possible by increasing the laser power. A 532 nm laser with 200 mW power (Boys et al., 2019) and a 785 nm laser with 250 mW power (Bergholt et al., 2017) have been reported to not damage cells in submerged microscopy systems. A too high laser power, however, can damage organic tissue and therefore there is a tradeoff between depth penetration and tissue integrity. Other Raman methods, such as Spatially Offset Raman Spectroscopy (SORS) and Transmission Raman Spectroscopy (TRS) were reported to give better signals for sub-surface spectra collection (Vardaki et al., 2015; Ghita et al., 2016; Matousek and Stone, 2016). Despite their applications for imaging other tissue such as bone (Matousek et al., 2006; Cui et al., 2020), to the best of our knowledge, these techniques have not been reported for cartilage imaging.

Additionally, although the non-negative spectral fitting was successful, it is important to note that the reference spectra were collected from dry forms of the biomacromolecules. As compared to dry forms, in hydrated cartilage tissue, biomacromolecules like collagen and GAGs have different tertiary and quaternary structures due to the hydration of these molecules and intermolecular interactions. For example, the Raman signature of collagen is strongly dependent on the size and orientation of collagen fibers, the aggregation of aggrecan can affect its spectra, and the hydration of chondroitin sulfate also alters the Raman signature (Bansil et al., 1978; Ellis et al., 2009; Masic et al., 2011; Galvis et al., 2013). In future work, obtaining hydrated reference spectra may yield more nuanced maps of macromolecule orientation and confirmation in the hydrated cartilage tissue. Some features of the Raman fingerprint area can also indicate detailed differences among compositions, such as chondroitin-4-sulfate (C4S) and chondroitin-6-sulfate (C6S) (Ellis et al., 2009). Nevertheless, more advanced analysis techniques are required to decompose spectra of similar molecules from the Raman spectra of the cartilage tissue.

This study collected data from the articular surface and on hydrated tissue. This technique has the potential to be incorporated into a fiber-optic Raman device, creating an optical fiber-based confocal Raman detection unit for an arthroscope (Esmonde-White et al., 2011; Bergholt et al., 2016a; Bergholt et al., 2017; Kandel et al., 2020). Since *in vivo* Raman spectra have been reported to be collected from human skin, lung, and bone (Matousek et al., 2006; Zeng et al., 2008), such a device may advance the analysis for *in vivo* animal models and diagnoses of early stage OA. Here, we have only applied this technique to a simplified enzymatic digestion model of bovine cartilage. Human cartilage, in contrast, is reported to be thicker in general but has a similar thickness in the superficial zone (Rieppo et al., 2003; Taylor et al., 2012). A higher composition of non-GAG solid content is also observed in human cartilage (Démarteau et al., 2006). Therefore, Raman maps that have different distribution or higher collagen content are expected to be acquired when human samples are studied, while superficial zone (50–100 μm thick for bovine cartilage) and transition zone (100–200 μm thick for bovine cartilage) of the samples (Mansfield and Winlove, 2017) can still be covered. Naturally occurring OA could be more complex, possibly including a roughened surface or thinner cartilage layer, which may result in lowered reflection or signal contribution from bone autofluorescence, leading to difficulties in analyzing Raman data. Other biomolecules, like hemoglobin and lipids, can also cause excessive background signals or distortions in Raman spectra, leading to further difficulties. This effect may prove challenging for *in vivo* imaging, where the bathing medium is synovial fluid that may contain components that complicate the interpretation of Raman data.

## 5 Conclusion

This work investigated the ability of confocal Raman microspectroscopy to determine the GAG distribution within trypsin-degraded bovine articular cartilage. We applied two different scan geometries using Raman microspectroscopy, cross-section scans and depth scans, on a trypsin digestion cartilage model for component analysis. The spatial distributions of major components (e.g., collagen, CS) within the articular cartilage obtained from Raman maps were qualitatively similar to the spatial distributions revealed by histology. Native bovine cartilage samples had lower GAG content at the articular surface, with an increase in GAG content at the deeper zone. Fully trypsin-digested samples show negligible GAG content throughout the entire tissue. We found that both scan geometries can provide similar Raman spectra when measuring beneath the articular surface. In cross-section scans, the data were collected from the exposed articular-surface-to-bone cut face. The depth scan enables depth-dependent spectra collection at the articular surface by moving the focal plane beneath the tissue surface, providing a data collection method that does not require sectioning of the tissue. In partially digested samples, zonal GAG depletion was detected by both scanning geometries. A thin digestion region at a ∼100 μm scale was observed. Regional compositional differences were shown both in point spectra and in Raman maps and recapitulate the results shown in histology. This work demonstrates that confocal Raman microscopy is capable of high-resolution compositional analysis for degraded articular cartilage. The results lay a foundation for non-invasive measurements of cartilage composition for *in vivo* studies and clinical early-stage OA diagnosis.

## Data Availability

The raw data supporting the conclusion of this article will be made available by the authors, without undue reservation.
